# Pressão Arterial em Crianças. O Papel Fundamental da Atividade Física e da Gordura Corporal

**DOI:** 10.36660/abc.20210117

**Published:** 2021-05-06

**Affiliations:** 1 Klaipeda University Faculty of Health Sciences Klaipeda Lituânia Klaipeda University - Faculty of Health Sciences, Klaipeda – Lituânia

**Keywords:** Pressão Arterial, Hipertensão/hereditariedade, Fatores de Risco, Criança, Obesidade, Sobrepeso, Atividade Física, Exercício, Sedentarismo, Saúde Pública

A hipertensão é o principal fator de risco global para doença renal crônica e doenças cardiovasculares além de ser a principal causa de morte prematura em todo o mundo.^1^ O número de adultos com pressão alta aumentou de 594 milhões em 1975 para 1,13 bilhão em 2015.^2^ A Organização Mundial da Saúde (OMS) estima que 1 em cada 4 homens e 1 em cada 5 mulheres tinham hipertensão e que, em 2025, 1,56 bilhão de adultos viverão com hipertensão.

O aumento da pressão arterial na infância está se tornando mais comum na população pediátrica em geral, representando um desafio considerável para a saúde pública em todo o mundo.^3^ Estudos têm sugerido que a hipertensão arterial na infância parece seguir desde a infância até a idade adulta^4^ e está associada a eventos cardiovasculares prejudiciais ao longo da vida.^3^ No entanto, a pressão arterial elevada é um dos mais importantes contribuintes evitáveis para doenças e morte e é considerada um dos principais fatores de risco modificáveis para doenças cardiovasculares com raízes na infância.^4^^.^^5^ Estudos indicam que níveis pressóricos elevados durante a infância são uma condição multifatorial.^6^ Genética, idade, sexo, etnia, sobrepeso/obesidade, ingestão de sódio e potássio, sedentarismo e fatores socioeconômicos foram apontados como os principais fatores de risco para hipertensão.^7^

A inatividade física e a obesidade se tornaram um problema de saúde global e as evidências indicam que ambas estão independentemente associadas ao aumento da pressão arterial.^8^^–^^10^ A prevalência da hipertensão infantil está aumentando paralelamente aos aumentos globais na prevalência de sobrepeso e obesidade.^8^ Além disso, a hipertensão relacionada à obesidade contribui ainda mais para o agrupamento de fatores de risco cardiometabólico.^8^

A atividade física e o alto comportamento sedentário desempenham um papel fundamental na saúde de crianças e adolescentes.^9^^,^^10^ A literatura atual relata que a atividade física confere benefícios para a melhoria da aptidão física (aptidão cardiorrespiratória e muscular), saúde cardiometabólica (pressão arterial, dislipidemia, glicose e resistência à insulina), saúde óssea, resultados cognitivos (desempenho acadêmico, função executiva), saúde mental (redução dos sintomas de depressão); e redução da adiposidade.^9^ Além disso, alguns estudos em crianças demonstraram que baixos níveis de aptidão cardiorrespiratória (ACR) estão inversamente associados ao aumento da pressão arterial.^11^^,^^12^ Assim como um alto nível de ACR na infância está associada a níveis normais de pressão arterial na idade adulta.^13^^,^^14^

De fato, a prevalência de níveis elevados de pressão arterial durante a infância também se tornou um problema de saúde pública significativo.^1^ Do ponto de vista da saúde pública, estudos confiáveis para identificar possíveis mecanismos associados ao aparecimento da hipertensão servem como base para prevenção e tratamento adequados, bem como para a alocação de recursos de saúde baseada em evidências e formulação de políticas. Levando tudo isso em consideração juntamente com a condição multifatorial da hipertensão arterial, do estudo publicado na ABC Cardiol,^15^ os autores buscaram investigar as associações entre medidas antropométricas, composição corporal, atividade física moderada-vigorosa e ACR com pressão arterial em crianças de 6 a 12 anos. Os autores observaram que a gordura corporal (porcentagem de gordura, índice de massa corporal e relação cintura/altura) estava negativamente associada aos níveis de pressão arterial. Além disso, observaram que a atividade física moderada-vigorosa e a ACR também tiveram grande impacto na pressão arterial. Os achados do estudo^15^ são particularmente importantes do ponto de vista da saúde pública, uma vez que tanto a atividade física quanto o peso corporal são fatores de risco modificáveis para a prevenção da hipertensão, ambos devem ser considerados simultaneamente em intervenções futuras. A identificação precoce de altos índices de gordura corporal e baixos níveis de ACR e atividade física na infância podem permitir intervenções precoces, prevenindo a hipertensão em idades precoces e adultas.

O estudo^15^ apresenta alguns pontos fortes importantes que devem ser destacados, como a novidade da análise do impacto de diversas variáveis sobre os níveis pressóricos em crianças de ambos os sexos, bem como a avaliação objetiva de atividade física com acelerômetros, uma vez que esses dispositivos não dependem da memória do sujeito e podem capturar todo o padrão diário de atividade física. O estudo também apresenta algumas limitações, como seu desenho transversal, e os autores não podem inferir que as associações observadas refletem relações causais. Além disso, faltam dados coletados sobre a ingestão alimentar, o que poderia fornecer um modelo preditor mais robusto.

Em conclusão, a pressão arterial elevada na infância representa um desafio considerável à saúde pública em todo o mundo. A hipertensão pediátrica é uma condição que tem efeitos profundos na vida adulta, aumentando o risco de eventos cardiovasculares futuros.^6^ Portanto, considerando que tanto a atividade física quanto a obesidade/sobrepeso são as principais condições modificáveis, interagindo com as alterações epigenéticas, elas devem ser consideradas simultaneamente em futuras intervenções com o objetivo de melhorar o perfil de saúde das crianças. O incremento da atividade física ou exercício para melhorar a aptidão física e diminuir a obesidade/sobrepeso pode ser uma estratégia preventiva eficaz para a redução e proteção contra o aumento da pressão arterial. No entanto, levando em consideração os resultados do estudo,^15^ bem como a literatura atual, especialistas científicos recomendam fortemente que crianças e adolescentes façam pelo menos uma média de 60 minutos por dia de atividade física de intensidade moderada a vigorosa, principalmente aeróbica, durante a semana para oferecer benefícios significativos à saúde e mitigar riscos. Além disso, atividades aeróbicas de intensidade vigorosa, bem como aquelas que fortalecem músculos e ossos, devem ser incorporadas pelo menos 3 dias por semana.
