# Supplementary material for: Pressão Arterial em Crianças. O Papel Fundamental da Atividade Física e da Gordura Corporal
Source: Arq Bras Cardiol. 2021 May 6;116(5):957–8. [Article in Portuguese] doi: 10.36660/abc.20210117 (PMC8121466; doi:10.36660/abc.20210117)
Supplement: Translation

# Translation: Blood Pressure in Children. The Key Role of Physical Activity and Body Fatness

**Affiliations:** 1 Klaipeda Lituânia Klaipeda University - Faculty of Health Sciences, Klaipeda – Lituânia

**Keywords:** Blood Arterial, Hypertension/heredity, Risk Factors, Child, Obesity, Overweight, Physical Activity, Exercise, Sedentarism, Public Health

High blood pressure is the leading global risk factor for chronic kidney disease and cardiovascular diseases and is the leading cause of premature death worldwide.^1^ The number of adults with high blood pressure increased from 594 million in 1975 to 1.13 billion in 2015.^2^ The World Health Organization (WHO) estimates 1 in 4 men and 1 in 5 women had hypertension and that by 2025, 1.56 billion adults will be living with hypertension.

Childhood raised blood pressure is becoming more common in the general pediatric population, representing a considerable public health challenge worldwide.^3^ Studies have suggested that childhood high blood pressure seems to track from childhood to adulthood^4^ and is associated with detrimental lifelong cardiovascular events.^3^ However, raised blood pressure is one of the most important preventable contributors to disease and death and is considered to be one of the major modifiable risk factors for cardiovascular disease with roots in childhood.^4^^,^^5^ Studies indicate that high blood pressure levels during childhood are a multifactorial condition.^6^ Genetics, age, gender, ethnicity, overweight/obesity, sodium and potassium intake, physical inactivity, and socioeconomic factors have been named as the main risk factors for hypertension.^7^

Physical inactivity and obesity have become a global health issue and the evidence indicate that both are independently associated with raised blood pressure.^8^^–^^10^ The prevalence of childhood hypertension is rising in parallel with global increases in the prevalence of overweight and obesity.^8^ In addition, obesity-related hypertension contributes further to the clustering of cardiometabolic risk factors.^8^

Physical activity and high sedentary behavior play a key role in children and adolescents health.^9^^,^^10^ Current literature report that physical activity confers benefits for improved physical fitness (cardiorespiratory and muscular fitness), cardiometabolic health (blood pressure, dyslipidemia, glucose, and insulin resistance), bone health, cognitive outcomes (academic performance, executive function), mental health (reduced symptoms of depression); and reduced adiposity.^9^ In addition, some studies in children have demonstrated that low levels of cardiorespiratory fitness (CRF) is inversely associated with raised blood pressure.^11^^,^^12^ As well as a high level of CRF in childhood is associated with normal levels of blood pressure in adulthood.^13^^,^^14^

Indeed, the prevalence of a high levels of blood pressure during childhood has also become a significant public health issue.^1^ From the public health perspective, reliable studies to identify possible mechanisms associated to hypertension onset serve as a basis for adequate prevention and treatment, as well as evidence-based health resource allocation and policy making. Taking all this into account along with the multifactorial condition of high blood pressure, the study of published in ABC Cardiol,^15^ the authors sought to investigate the associations between anthropometric measurements, body composition, moderate-vigorous physical activity, and CRF with blood pressure in children aged 6 to 12 years old. The authors found that body fatness (percentage of fat, body mass index, and waist/height ratio) was negatively associated with blood pressure levels. In addition, they observed that moderate-vigorous physical activity and CRF, also had a great impact on blood pressure. Findings from study^15^ are particularly important from a public health perspective, since both physical activity and body weight are modifiable risk factors for the prevention of hypertension, both should be simultaneously considered in future interventions. The early identification of high body fatness, and low levels of CRF and physical activity in childhood may allow early interventions, thereby preventing hypertension at an early age as well as in the adulthood.

The study^15^ present some important strengths that should be highlighted, such as the novelty of the analysis of the impact of several variables on blood pressure levels in children of both genders as well as the objectively assessment of physical activity with accelerometers, as these devices do not rely on subjects’ recall and may capture the entire daily pattern of physical activity. The study also presents to certain limitations, such as its cross-sectional design, and the authors cannot infer that their observed associations reflect causal relationships. Moreover, there is a lack of data collected regarding food intake, which could provide a more robust predictor model.

In conclusion childhood raised blood pressure represents a considerable public health challenge worldwide. Pediatric hypertension is a condition that has profound effects on later life, increasing the risk for future cardiovascular events in adulthood.^6^ Therefore, considering that both physical activity and obesity/overweight are main modifiable conditions, interacting with epigenetic changes, they should be simultaneously considered in future interventions aiming to improve the health profile of children. The increment of physical activity or exercise to improve physical fitness and decrease obesity/overweight may be an effective preventive strategy for reduction and protection against raised blood pressure. However, taking the findings of study^15^ into account, as well as the current literature, scientific experts strongly recommend that children and adolescents should do at least an average of 60 minutes per day of moderate to vigorous-intensity, mostly aerobic, physical activity, across the week to offer significant health benefits and mitigate health risks. In addition, vigorous-intensity aerobic activities, as well as those that strengthen muscle and bone, should be incorporated at least 3 days a week.
